# A Case Report on Mandibular Metastasis From a Breast Carcinoma

**DOI:** 10.7759/cureus.29781

**Published:** 2022-09-30

**Authors:** Rishwanth Vetri, Vinni Anna Jacob, Vishmita Kannichamy, Surabhi Sainath

**Affiliations:** 1 Department of General Surgery, Stanley Medical College and Hospital, Chennai, IND

**Keywords:** mammography, distant metastases, lymphadenopathy, mandibular metastasis, breast carcinoma

## Abstract

Despite the rise in the number of cases of breast cancer in recent years, clinical diagnosis of a primary tumor in cases presenting with metastasis to the oral cavity poses a challenge in modern medicine because of its rare presentation.

We report a case of breast cancer which presented as a painless swelling in the jaw. A 37-year-old multiparous woman consulted her dentist with complaints of toothache and swelling over the right cheek. On examination, she was diagnosed with dental caries and an orthopantomogram (OPG) was done to evaluate the swelling which revealed an area of rarefaction with an irregular margin on the right angle of the mandible. With suspicion of malignancy, the head, neck, oral and pharyngeal regions were thoroughly inspected and palpated. An ultrasonogram (USG) of the neck was done, which was normal and a core needle biopsy of the oral swelling was performed which showed metastatic carcinomatous deposits with pan-cytokeratin (PAN-CK) positivity, estrogen receptor (ER) positivity, and the Ki-67 value was 10% to 20% which was suggestive of breast carcinoma metastasis. Thereafter, the patient was referred for a surgeon’s opinion. A breast examination was then done which revealed a lump in the right breast with a retracted nipple. A core needle biopsy of the lump revealed that it was ER and progesterone receptor (PR) positive and human epidermal growth factor receptor 2 (HER-2/neu) negative which confirmed the clinical diagnosis of breast cancer.

Since the incidence of oral metastatic tumors is low, the likelihood of an early diagnosis of the distant primary tumor is reduced. Hence, all lesions of the oral cavity should be evaluated with due diligence considering the possibility of it being secondary metastases from distant tumors.

## Introduction

Despite the advances in diagnosis and treatment, carcinoma of the breast has become one of the leading causes of death among women worldwide, especially in developed countries [1]. In India, around 6% to 25% of breast cancer patients initially present with symptoms of metastatic disease before their diagnosis [2]. These women do not seek early medical attention because of poor awareness, lack of effective screening in rural areas, social taboos, and financial hardships. Breast cancer can also be latent where the metastases appear years after treatment of the primary tumor or it can be occult breast cancer which is an uncommon condition where there are nodal or distant metastases without evidence of the primary breast tumor [3]. The presence of metastasis is highly important in determining the patient’s prognosis and mode of treatment. Early diagnosis aids the physician in timely intervention and to choose the best available treatment.

Metastasis in breast cancer is commonly seen in the lungs, liver, bones, pleura, brain, and kidneys and is rarely seen in adrenal glands and ovaries [4]. Metastasis to the oral cavity is not common and is seen in only around 1% of the cases [5-7]. In the oral cavity, the mandible is the most common site for metastasis [6-9]. Hence, diagnosis of the primary tumor becomes difficult because of this uncommon presentation. 

Here, we outline a rare case report of a patient with mandibular metastatic lesions which was noticed before identifying the primary tumor in the breast. Hormone receptors namely, estrogen receptor (ER), progesterone receptor (PR), human epidermal growth factor receptor 2 (HER-2/neu), and proliferation marker Ki-67 were the basic molecular markers utilized for diagnosis of this case. These markers along with additional investigations helped in determining the aggressiveness of the tumor and treatment for the patient.

## Case presentation

**Figure 1:**
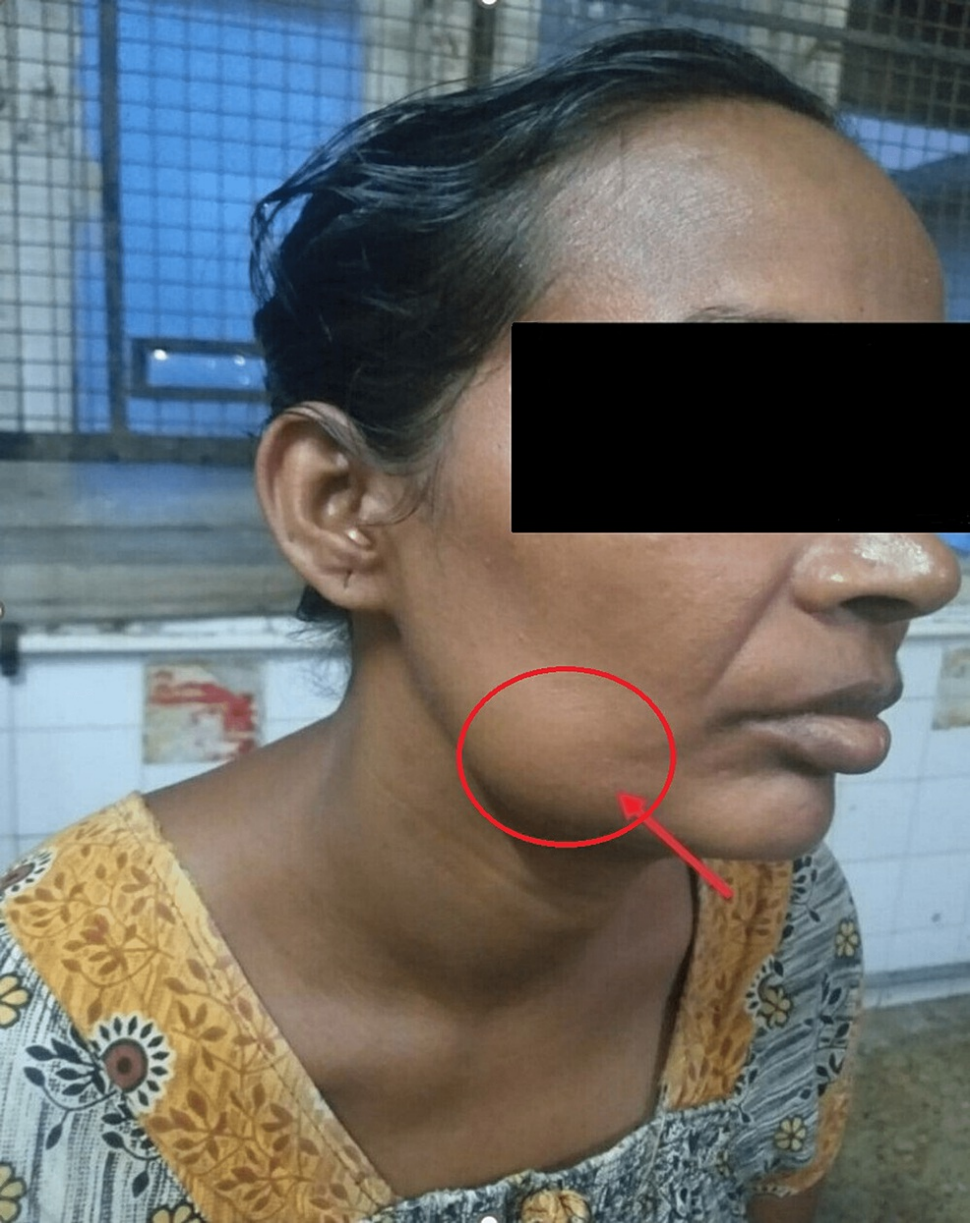
Clinical photo of the right mandibular swelling; the red arrow and circle show a 4 cm by 3 cm ovoid swelling on the right mandible.

A 37-year-old woman consulted her dentist with chief complaints of toothache and painless swelling over the lower aspect of the right cheek which had begun seven months back. After thorough inspection and palpation of the head, neck, oral cavity, and pharynx, a 4 cm by 3 cm ovoid, bony hard, non-tender swelling over the angle of the right mandible was noted extending 5 cm from the tragus (superiorly), 4 cm from the hyoid bone (inferiorly), 4 cm from the angle of the mouth (medially) and 2 cm from the right anterior border of the sternocleidomastoid (laterally) (Figure 1). She was also diagnosed with dental caries for which tooth extraction was done and further evaluation was done for the mandibular swelling.

**Figure 2:**
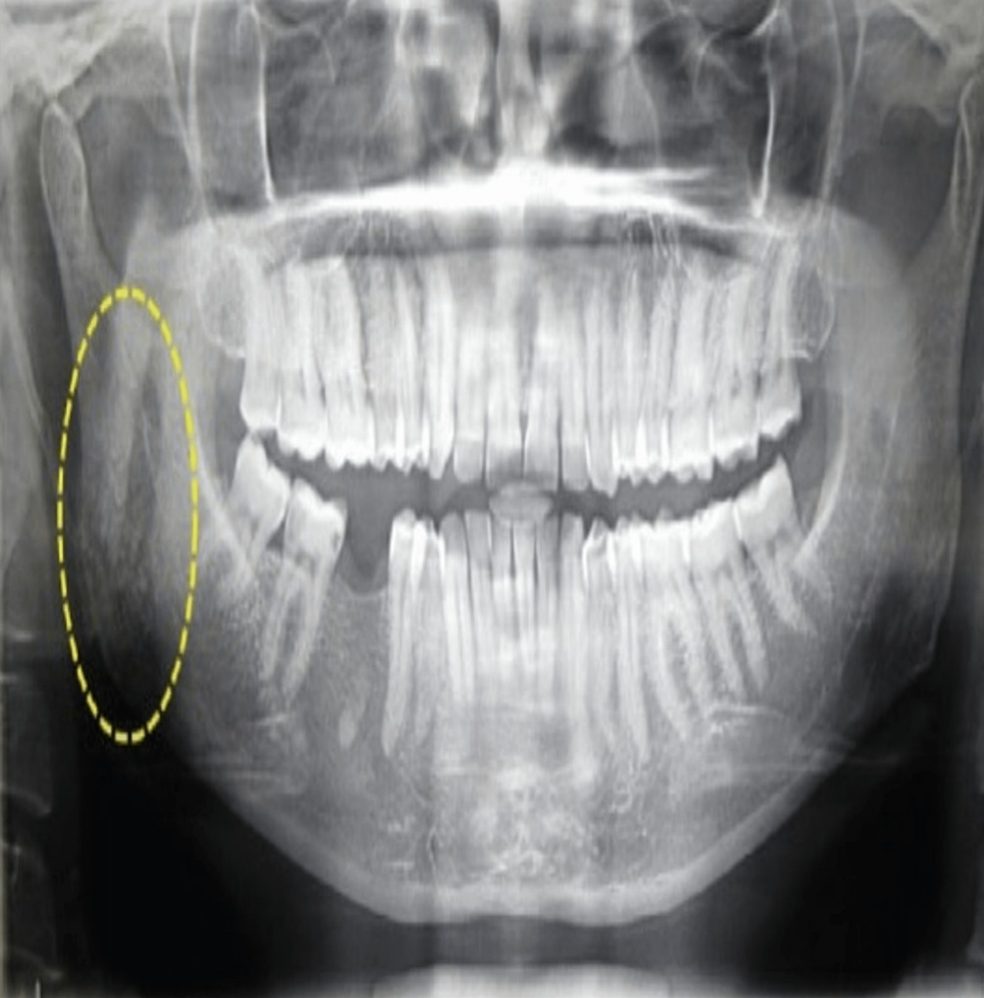
Orthopantomogram of the patient, yellow circle shows the area of rarefaction.

On evaluation, the orthopantomogram (OPG) revealed an area of rarefaction with an irregular margin on the right angle of the mandible (Figure 2). Ultrasonography (USG) bilateral neck done as an investigation for primary head and neck tumors was normal; there was no significant lymphadenopathy.

**Figure 3:**
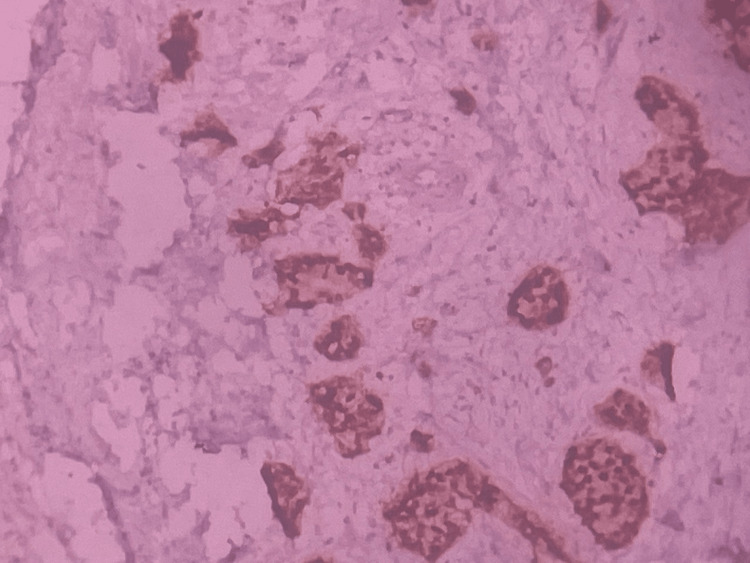
ER-staining (IHC) showing positivity in the tumor cells of the right mandibular swelling. ER: estrogen receptor; IHC: immunohistochemistry

With suspicion of a primary mandibular tumor, a core needle biopsy was performed, which showed metastatic carcinomatous deposits with pan-cytokeratin (PAN-CK) positivity and ER positivity; Ki-67 value was 10% to 20% which was suggestive of metastatic deposits from a primary tumor in the breast (Figure 3). Hence, the patient was referred for a surgeon’s opinion.

Because of PAN-CK and ER positivity, breast cancer was suspected and a detailed history was taken from the patient which revealed that the patient had right nipple retraction for the past eight months. The patient had no family history of breast and ovarian malignancies and no risk factors including radiation exposure in the past, consumption of oral contraceptive pills, or any history of hormone replacement therapy. She was not a smoker and she had no history of alcohol consumption. She had a healthy lifestyle and was moderately built and nourished. She reached menarche at the age of 13, she had regular cycles not associated with menorrhagia or dysmenorrhea. She had her first child at the age of 15, and second child at the age of 17, and breastfed them for a total of 21 months. 

**Figure 4:**
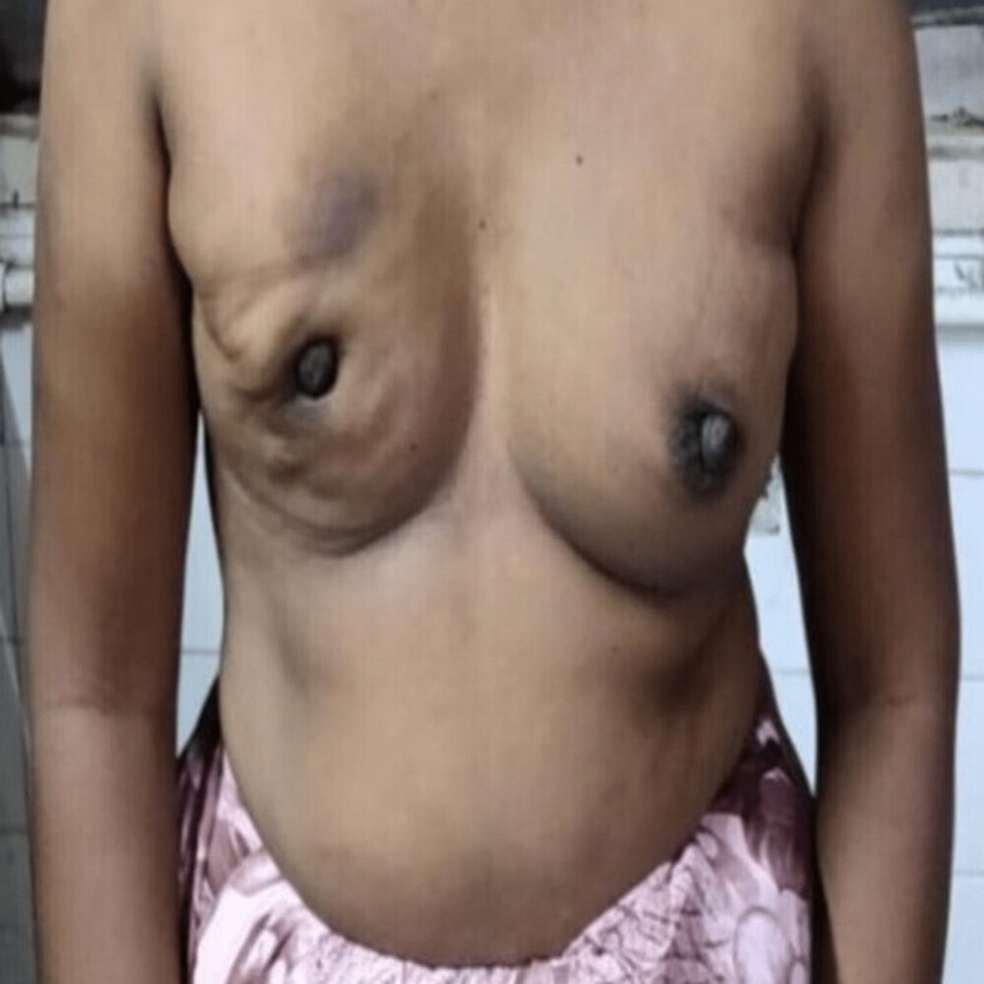
Clinical photo showing retro areolar lump and circumferential nipple retraction in the right breast. Puckering is also seen from 4 o'clock to 10 o'clock position in the right breast.

A breast examination was done which revealed a palpable and irregular-shaped 2 cm by 2 cm retro-areolar lump in the right breast with a retracted nipple, hard in consistency with irregular margins (Figure 4). Puckering was also seen in the lower inner, lower outer, and upper outer quadrants of the right breast. A 1 cm by 1 cm mobile, non-tender and palpable central right axillary lymph node was also present. Clinical examination of the left breast and other systemic examinations was normal. 

**Figure 5:**
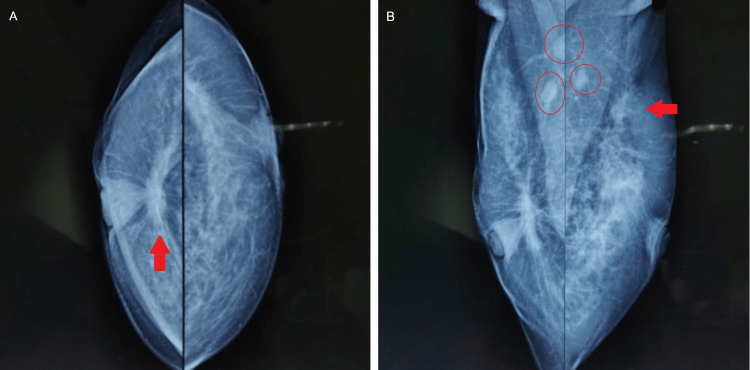
X-ray mammogram of bilateral breasts with the axilla. Arrow (panel A) shows the retro areolar lesion in the right breast. Arrow (panel B) shows the hypoechoic lesion in the left breast. Circles (panel B) shows bilateral lymphadenopathy. A: craniocaudal view. B: mediolateral oblique view

Mammography was then done which showed spiculated opacity in the retro-areolar region with diffuse skin thickening in the right breast and irregular spiculated hypoechoic lesion noted at the 2 o’clock position in the left breast (Figures 5A-5B). Bilateral axillary lymphadenopathy was also present.

A core needle biopsy of the right breast lump diagnosed the lesion as invasive ductal carcinoma - no specific type (NST). The lump was ER and PR positive, HER-2/neu negative, and the Ki-67 value was less than 10%. The right axillary node was not biopsied. An image-guided core needle biopsy of the left breast was done; the results were positive for malignancy, the left breast lesion was also diagnosed as invasive ductal carcinoma - NST. The receptor status of the left breast lesion was found to be ER positive, PR positive, HER-2/neu negative, and the Ki-67 value was less than 10%. An ultrasound-guided fine needle aspiration cytology (FNAC) of the left axillary node showed that the metastatic carcinomatous deposits were probably from ductal carcinoma of the breast. BRCA1 and BRCA2 gene testing could not be done as the facilities required for the same were unavailable at our institution. USG of the abdomen showed no significant abnormality.

**Figure 6:**
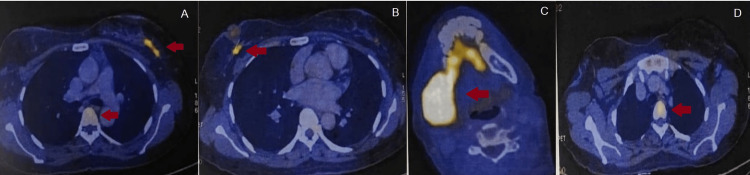
PET-CT showing metabolically active areas (red arrows). A: hypermetabolic lesion seen in the left breast and D5 vertebra. B: hypermetabolic lesion seen in the retro areolar region in the right breast. C: hypermetabolic lesion seen in the right mandible. D: hypermetabolic lesion seen in D1 vertebra. PET-CT: positron emission tomography-computed tomography

A positron emission tomography-computed tomography (PET-CT) scan showed metabolically active soft tissue density in the retro areolar aspect of the right breast and upper outer quadrant of the left breast, confirming the diagnosis (Figures 6A-6D). Few metabolically active right-sided level I axillary lymph nodes and few left-sided level I and II axillary lymph nodes were also detected. Metabolically active lytic lesions were also seen in the right parietal bone, right ramus of mandible, right first rib, left seventh rib, and D1 and D5 vertebra, medial end of the clavicle, sacrum, right iliac bone, and proximal shaft of the right femur.

**Figure 7:**
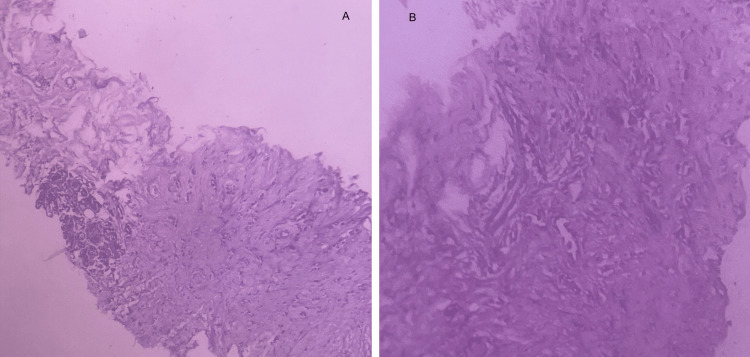
Histopathology of right breast (panel A) and left breast (panel B) showing similar histology and grade of differentiation.

Using Chaudary and Millis clinicopathological criteria [10], the left breast lesion was found to be a metastasis from the right breast carcinoma as biopsy of both left and right breast showed that the second tumor in the left breast was histologically the same as the tumor in the right breast, had similar histological grade and there was also evidence of local and distant metastasis (Figures 7A-7B). 

Hence, a final diagnosis of bilateral breast carcinoma with bilateral axillary lymphadenopathy with multiple metastases was made. Cancer staging done according to the Tumor, Nodes, Metastases (TNM) staging system was cT4aN1M1 - stage IV. After obtaining the tumor board’s opinion, the patient was given external beam radiotherapy, a total dose of 30 Gy given as 10 fractions for the mandibular swelling.

Following radiotherapy, the patient was started on palliative chemotherapy in July 2019 with adriamycin, cyclophosphamide, and zoledronic acid (palliative care for bony metastasis) (Table 1). After three cycles of chemotherapy, clinically, there was a subjective reduction of 50% in the tumor size and a reduction of 80% after six cycles. Since the tumor burden was reduced and because of the patient's hormone receptor status, chemotherapy was discontinued after six cycles, and hormone therapy was started; the patient was put on tamoxifen along with zoledronic acid (Table 1). After one month of hormone therapy, the patient defaulted treatment. The patient returned after six months and hormone therapy was restarted. After 18 months of therapy, zoledronic acid was discontinued and the patient again defaulted treatment for three months. She was prescribed tamoxifen on return, which was continued till December 2021 when it was found that the patient's hormone levels (serum follicle-stimulating hormone, serum luteinizing hormone, and serum estradiol values) were within the post-menopausal range. Hence tamoxifen was discontinued and the patient was prescribed letrozole and calcium (to prevent osteoporosis). A USG breast done in August 2022 showed no evidence of any focal lesions. The current plan of treatment is to continue letrozole but if there is any tumor progression or unacceptable toxicity, exemestane/fulvestrant with cyclin-dependent kinase 4/6 (CDK 4/6) inhibitors (abemaciclib/palbociclib/ribociclib) is to be given.

**Table 1:** Timeline of palliative chemotherapy and hormone therapy.

DATE	TREATMENT	
July - December 2019 (CYCLE 1-6)	Injection Adriamycin 80 mg in 10 ml normal saline given over 15 minutes. Injection Cyclophosphamide 800 mg in 500 ml normal saline given over 2 hours. Injection Zoledronic Acid 4mg in 100 ml given over 30 minutes.	
January 2022	Tablet Tamoxifen 20 mg OD. Injection Zoledronic Acid 4 mg in 100 ml given over 30 minutes.	
Patient defaulted treatment for 7 months		
October 2020 –March 2021	Tablet Tamoxifen 20 mg OD. Injection Zoledronic Acid 4 mg in 100 ml given over 30 minutes.	
April 2021	Zoledronic Acid discontinued after 18 months of treatment. Tablet Tamoxifen 20 mg OD.	
Patient defaulted treatment for 3 months		
July 2021 – December 2021		Tablet Tamoxifen 20 mg OD
January 2022 – till present		Tablet Letrozole 25 mg OD

## Discussion

Oral cavity metastases account for only 1% of all oral malignancies; these metastases to the oral cavity are commonly from the breast, lungs, and kidneys [5]. In the oral cavity, the mandible is the most common site for metastasis [6-9]. Differential diagnosis of mandibular swelling includes lesions resulting from trauma, infections, and metabolic processes, as well as benign and malignant tumors [11]. Tumors of the mandible can either arise primarily from the bone itself or can arise through mandibular invasion from adjacent tumors of the oral cavity and sinuses or metastatic tumors. Primary tumors of the mandible include ameloblastoma, ossifying fibroma, osteogenic sarcoma, reticulum cell sarcoma, chondrosarcoma, myxosarcoma, epidermoid carcinoma, adenocarcinoma, and giant cell sarcoma. Mandibular invasion of squamous cell carcinoma is the most frequently encountered carcinoma of the jaw and is associated with a poor prognosis. Squamous cell carcinoma of the oral cavity is diagnosed mainly through CT, MRI, and orthopantomography [12].

Breast carcinoma can spread locally as well as cause distant spread through lymphatics and the bloodstream. Breast cancer metastases commonly spread to the lungs, liver, bones, pleura, and kidneys [4]. The carcinoma spreads to the mandible through the bloodstream as the jaw bone does not have lymphatic drainage [13].In females, the majority of the mandibular metastasis is from the breast [6,7]. There have been case reports of mandibular metastasis in breast cancer in men, although male breast cancers are sporadic compared to females [4,14]. Metastasis can even appear in patients after a prolonged disease-free period [15]. Once the carcinoma spreads to the oral cavity, the presenting symptoms are numerous and varied, which include bleeding, swelling, loose tooth, numbness, trismus, foul-smelling breath, pathological fractures, ulceration, and lymphadenopathy [15,16]. A review of articles showed that numb chin syndrome (NCS) is also a sign of oral metastasis. NCS is a condition affecting the areas supplied by mental or inferior alveolar nerves, it is marked by hypoesthesia, paraesthesia, and infrequently by pain over the chin [17]. Since these symptoms are characteristic of other dental diseases and also because there are varied presentations of oral cavity metastasis, they can be misdiagnosed.

Oral metastasis can also be the first presentation before the primary tumor is diagnosed, for instance, in this case, breast carcinoma was diagnosed after the appearance of the mandibular swelling. At the time of diagnosis of the metastasis in the oral cavity, there can also be other sites of distant metastatic spread indicating an advanced stage. In this case, cancer had already spread to the parietal bone, ribs, vertebrae, clavicle, sacrum, iliac bone, and femur at the time of diagnosis. Oral cavity metastatic lesions are associated with a poor prognosis, which might be fatal. Once diagnosed, less than 10% of the patients survive for more than four years [18]. Since breast carcinoma will be at an advanced stage when there are oral metastases, the primary management is palliative therapy.

This case also draws attention to the fact that the patient did not know that nipple retraction was an abnormal sign which required medical attention and was unaware that nipple retraction could be a possible sign of carcinoma. The patient could have also been in denial or could have delayed consultation with a doctor since it was a painless condition that was not visible to the public eye, whereas the mandibular swelling was visible and would have raised concern among family members which pushed her to visit a doctor, although the mandibular swelling appeared later than the nipple retraction. This stems from a possibility of ignorance and lack of awareness of the disease. This ignorance points toward the inadequate reach of public education about breast cancer in remote villages and communities. Social determinants of health like lack of education, poverty, stigma, casteism, and racism are also contributing factors to disparities in the healthcare system. Despite continued attempts, the social gap in the healthcare system persists. This social gap discourages and prevents the deprived population from getting the same quality of healthcare as the affluent [19].

Even though national-wide screening programs are more effective than breast-self examination (BSE) since it has not shown any improvement in mortality rates, in countries with vast populations and disparities in healthcare systems like India, BSE is still recommended as it is a low-cost method that can be done anywhere and by everyone. In a study conducted among 200 women in rural India by Veena K et al., it was found that 75.5% of the women had an inadequate understanding of breast cancer and 80% of the women did not know about BSE [20]. Hence effective public education, national screening programs, and eliminating the disparities in the healthcare system will help in making women competent in self-diagnosing breast cancer and seeking medical attention.

The limitation of this case was the inability to do BRACA1 and BRACA2 gene testing due to inadequate facilities available at our institution.

## Conclusions

This case focuses on the significance of early evaluation of suspicious oral lesions even in the absence of significant history for a timely diagnosis of primary tumors. All physicians should be familiar with all the atypical presentations of breast carcinoma metastases to avoid wrong diagnosis and treatment. A detailed ‘triple assessment’ should be done to rule out breast carcinoma in all doubtful cases of oral swellings and lesions. Better awareness regarding breast cancer should also be raised, especially among illiterate women from socially and economically backward areas.
